# Classification of Citrus Diseases Using Optimization Deep Learning Approach

**DOI:** 10.1155/2022/9153207

**Published:** 2022-02-10

**Authors:** Ahmed Elaraby, Walid Hamdy, Saad Alanazi

**Affiliations:** ^1^Department of Computer Science, Faculty of Computers and Information, South Valley University, Qena, Egypt; ^2^Department of Math and Computer Science Faculty of Science, Port Said University, Port Fuad, Egypt; ^3^Department of Computer Science, College of Computer and Information Sciences, Jouf University, Sakakah, Saudi Arabia

## Abstract

Most plant diseases have apparent signs, and today's recognized method is for an expert plant pathologist to identify the disease by looking at infected plant leaves using a microscope. The fact is that manually diagnosing diseases is time consuming and that the effectiveness of the diagnosis is related to the pathologist's talents, making this a great application area for computer-aided diagnostic systems. The proposed work describes an approach for detecting and classifying diseases in citrus plants using deep learning and image processing. The main cause of decreased productivity is considered to be plant diseases, which results in financial losses. Citrus is an important source of nutrients such as vitamin C all around the world. On the contrary, citrus diseases have a negative impact on the citrus fruit and quality. In the recent decade, computer vision and image processing techniques have become increasingly popular for the detection and classification of plant diseases. The suggested approach is evaluated on the citrus disease image gallery dataset and the combined dataset (citrus image datasets of infested scale and plant village). These datasets were used to identify and classify citrus diseases such as anthracnose, black spot, canker, scab, greening, and melanose. AlexNet and VGG19 are two kinds of convolutional neural networks that were used to build and test the proposed approach. The system's total performance reached 94% at its best. The proposed approach outperforms the existing methods.

## 1. Introduction

The essential techniques of attaining the greatest economic value of citrus are the identification and categorization of citrus diseases. Citrus disease classification, as the most significant element of citrus disease processing, is progressively performed by machine learning than manual techniques such as computer image processing, pattern recognition, and other technologies. Automatic fruit classification using machine vision can not only solve difficulties such as poor productivity and inconsistent classification standards that come with human sorting but can also increase classification accuracy [1]. For many people in the world, agriculture has been their primary source of income. Agriculture's increased commercialization has had a significant impact on our environment. One of the most pressing problems in agriculture is the identification of plant diseases. Early disease identification helps in the prevention of disease transmission to other plants that leads to significant economic losses. Plant diseases have a wide variety of effects, from minor symptoms to full plantation loss, all of which have a significant influence on the agricultural economy [2].

Deep learning is now widely utilized in a variety of disciplines, including object identification [3], signal and speech recognition [4], biomedical image classification [5, 6], and segmentation [7]. Deep learning is also being utilized extensively in agriculture for the identification and categorization of plant diseases [8]. Convolutional neural network (CNN) is regarded as the most effective deep learning approach [9]. Several CNN architectures such as AlexNet [10], GoogLeNet [11], and others are utilized to identify and classify plant diseases. Furthermore, there are many researchers who used deep learning models for the identification and classification of citrus diseases (Pourreza et al. [12], Barman and Ridip [13], Xiaoling et al. [14], and Zia Ur Rehman et al. [15]).

The available dataset for training deep learning models has a significant impact on their performance. On the sufficiently big dataset, these models exhibit improved outcomes and excellent generalizability. The datasets currently available for citrus plant diseases usually lack sufficient images in a variety of situations, which are required for developing high-accuracy models. Given the small dataset, the model may overfit and perform poorly on the real-world test dataset. To improve the dataset, different data augmentation techniques such as affine transformation and perspective transformation are utilized [16]. Generative adversarial networks (GANs) are used to create counterfeit images when the training images are inadequate, and there is no ability for image manipulation techniques to change the outputs.

The major goal of this research is to use deep learning approaches to identify citrus plant diseases at a lower cost. Two distinct citrus images, fruit disease image (FDI) and leaf Disease image (LDI), are used to solve this problem. Viruses, fungi, mold, bacteria, and mites are the most common causes of diseases in plants. The proposed approach detects and classifies the affected plant's diseases and then presents the results in multiperformance metrics to prove the effectiveness of our models. When compared to prior or current methodologies, the proposed approach yields a result with less calculation time and more accuracy. We used stochastic gradient descent with momentum to optimize the models.

## 2. Related Work

Many approaches for detecting and classifying fruit diseases have been proposed by researchers in the fields of computer vision and machine learning [17]. For segmentation of arecanut bunches, Dhanesha et al. [18] employed the YCR color model approach. For segmenting bunches, the approach employs volumetric overlap error and dice similarity coefficient to estimate the similarity between the input image and ground truth. This technique focuses on segmenting arecanut branches that are not arecanut. The same approach is expanded using the HSV color model for the purpose of bunch segmentation [19].

To diagnose rice plant diseases, Ghosal and Sarkar [20] presented a VGG16 with transfer learning. The authors utilized four classes of images to train this classification, and VGG16 has an accuracy of up to 92.4%. Kumar et al. [21] presented a system to identifying diseases at coffee leaves, and radial basis function neural network, fuzzy logic-based expert system, transfer learning techniques, and CNN with data augmentation were used for identification. This study employs two types of datasets: original leaf images and chosen symptomatic portions from leaf images. There are five different kinds of leaf images in each dataset. The model performed well with a score of 97.61%.

For identifying the diseases in millet crops, Coulibaly et al. [22] used a VGG16 model using a transfer learning method. This study gathered 124 leaf images and divided them into two categories: mildew infections and healthy leaves. The accuracy of the VGG16 model was 95%.

A convolutional neural network was proposed by Hari et al. [23] as an effective method for detecting diseases in plant types such as grape, maize, tomato, and apple. The dataset comprises a total of 15,210 leaf images divided into ten classes, which were used to train and test the model. The accuracy of the suggested convolutional neural network was 86%.

For identifying diseases in tomato leaf images, Jiang et al. [24] employed a CNN model ResNet50. The collection contains 3,000 images that belong to three classifications. This model has 98.0% accuracy. For identifying diseases in plant leaves, Nandhini and Bhavani [25] offered machine learning methods such as KNN, decision trees, and SVM. To segment the diseased part of the leaf image, they employed a feature extraction technique that involves several steps, including converting RGB images to lab color space models for color feature extraction, K-means clustering, fast Fourier transform, and histogram, scale-invariant feature transform for shape feature extraction, and principal component analysis for lowering vector size. For classification, the algorithms discussed above were utilized, with SVM outperforming the other two techniques. Panchal et al. [26] utilized a random forest classifier to detect early blight, late blight infections, and bacterial spot in leaves of plants. The herpes simplex virus (HSV) method was utilized to separate the sick and healthy portions of the leaf in image segmentation, and a gray level co-occurrence matrix was used for feature extraction. Those models have a 98% accuracy rate.

Automatic plant disease identification for 28 distinct classes gathered from 15 different plants was presented by Kamal et al. [27]. A total of 23,352 images were chosen, ranging in size from 43 to 1,760 per class. For feature extraction, six distinct models were chosen: DenseNet 121, DenseNet 169, VGG19, InceptionV3, NasNetmobile, MobileNet, and ResNet. 50,000 images were gathered from 14 distinct crops by Agarwal et al. [28]. All the images were created using the same dimensions. Three convolutional layers with three max-pooling layers and various filters are included in the proposed model. Adhikari et al. [29] presented a methodology for automatically detecting plant illness, particularly in tomato plants, using image processing. The datasets such as gray spot, late blight, and bacterial canker included some images of three forms of tomato plant diseases. Some of the images were taken from the Internet, while others were taken with camera equipment on the premises. The CNN model that was formed contains 24 convolutional layers and two fully linked layers.

Janarthan et al. [30] created a system for the categorization of four distinct citrus leaf diseases that included an embedding module, a patch module, and a deep neural network. This study employed a dataset of 609 images. The patch module divides the various patches of lesion found on leaves into individual pictures, increasing the amount of dataset available for training. Background removal and data augmentation are among the preprocessing techniques employed. The training is performed with the deep Siamese network. The suggested approach achieved 94.04% with a minimal computing cost; adjusting roughly 2.3 million parameters are required to train the network. Pan et al. [31] described a deep convolutional neural network-based technique. Black spot, anthracnose, sand rust, canker, scab, and greening (HLB) are among the diseases included in the collection, which has 2,097 images. To expand the quantity of datasets available for training, data augmentation approaches are used. The dataset is partitioned into 6 : 2:2 for training, validation, and testing, correspondingly. Features are extracted and classified by using the DenseNet model. The last dense block in this work is changed to simplify the DenseNet model. With a decent forecast time, the proposed strategies achieved an accuracy of 88%.

Zhang et al. [32] proposed a technique for detecting canker disease. Deep neural networks are used in both rounds of this process. GANs (Generative adversarial networks) are employed in the initial stage to magnify the dataset by reproducing the original dataset and creating synthetic pictures. The second step is based on AlexNet, and it involves making modifications to the optimization target and updating the parameters via Siamese training, with an accuracy of 90.9% and a recall of 86.5%.

In this paper, we study and evaluate the effectiveness of several first-order optimizers, particularly for identifying images of citrus diseases using pretrained models such as AlexNet and VGG19. The pretrained AlexNet model surpasses the other architectures when it comes to categorising photos of citrus diseases, according to the findings of the experiments.

## 3. Proposed Methodology

**Figure 1:**
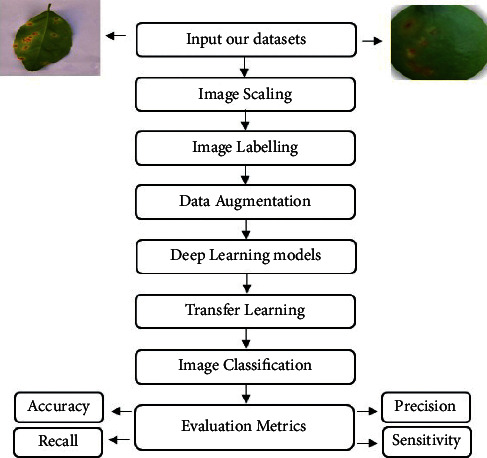
General architecture of our deep learning models.

In recent years, citrus plant diseases' automatic detection has grown in popularity by using deep neural networks. We present a short description of the proposed framework that will be used for detection and classiﬁcation of diseases in citrus plants using deep learning and image processing. The general architecture of our suggested deep learning models is depicted in Figure 1, which includes input datasets, preprocessing phase, deep learning model phase, transfer learning phase, classification phase, and evaluation metrics phase. First, it is possible to identify lesion patches on citrus fruits and expose them by using the suggested deep learning models. The second step is to classify citrus diseases. It takes a long time to train a neural network from scratch. It necessitates the use of a good hyperparameter selection technique. Instead, transferring the weights from a conventional pretrained network is simple and delivers better categorization performance measures.

**Figure 2:**
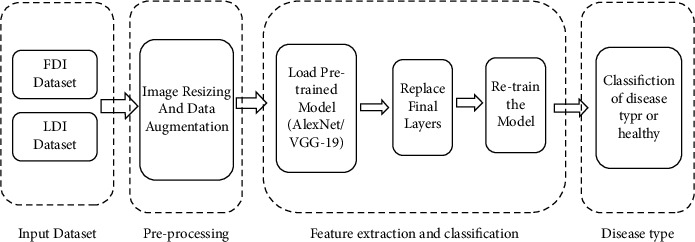
Transfer learning-based classification.

The schematic diagram of transfer learning-based citrus plant classification from the image of the diseases is shown in Figure 2. The disease images are scaled to fit into a pretrained network's standard input size. Data augmentation utilizing rotation is accomplished in the preprocessing step since a deep neural network works effectively with a higher number of images. The selected model's starting layers and network weights are transferred. The discriminative features from the illness images are extracted by the appropriate network. Modifying the last layers allows for classification.

### 3.1. Transfer Learning

A common deep learning approach is based on transfer learning in which pretrained model weights are transferred to a new classification issue. As a result, training becomes more efficient and easier. The architectures AlexNet and VGG19 are employed in this study. AlexNet [33] is made up of eight layers that may be learned (five convolutional and three fully connected layers). Due to its deep architecture, VGG19 [34] is a well-known pretrained model for image classification that works well. It has 47 layers, including sixteen convolutional layers, five maximum pooling layers, and three fully linked layers.

### 3.2. AlexNet

AlexNet [35] is made up of eight layers, five of which are convolutional and three of which are completely connected. Each convolutional layer is paired with a maxpooling layer and a normalization layer to reduce the image size and normalize the output pixel values [36]. For AlexNet, the images are resized to 224 ^∗^ 224 pixels. The first convolutional layer uses an input image of 224 ^∗^ 224 ^∗^ 3 pixels with 96 kernels of size 11 ^∗^ 11 ^∗^ 3 pixels and a stride of 4 ^∗^ 4. Here, 3 denotes creating three-channel RGB images. This layer has a total of 34,944 parameters. The maxpooling layer follows with a pool size of 2 ^∗^ 2 and 2 strides. The second convolutional layer uses data from the preceding layer and has a 256-kernel size for 1 stride, followed by maxpooling layers with 2 ^∗^ 2 pool size and 2 strides. This layer has a total of 2,973,952 parameters. The third convolutional layer has a kernel size of 384 with 1 stride and is followed by a maxpooling layer with a pool size of 22 with 2 strides, which takes data from the previous layers. This layer has a total of 885,120 parameters. The fourth convolutional layer, with a kernel size of 384 and 1 stride, takes input from the preceding layers. This layer has a total of 1,327,488 parameters. The fifth convolutional layer has 256 parts and 1 step and is followed by a maxpooling layer with 22 pool sizes and 2 steps, which integrates input from previous layers. The total parameter is now 884,992. There are three thick layers with 4,096 neurons after the five convolutional layers. There are a total of 28,079,671 parameters utilized. The activation function utilized here is the Relu, while the Softmax activation function is used for the last dense layer.

### 3.3. VGG19

This VGG19 [34] network is identical to VGG16, but instead of 16, it will have 19 layers, including 16 convolutional layers and three entirely linked dense layers. The first and second layers each include 64 filters and 3 ^∗^ 3 kernels, which are followed by the maxpooling layer. Following the maxpooling layer, there are 128 filters with a 33% kernel in the second and third convolutional layers, and following that there are four convolutional layers with 256 filters of 3 ^∗^ 3 kernel and a maxpooling layer in that order. Two further convolutional layers with 512 filters of 3 ^∗^ 3 kernel are put in sequence, followed by a maxpooling layer. This output is then routed into layers that are fully coupled. With 4096, 4096, and 1000 neurons, there are three fully connected thick layers. For all layers, the activation function is Relu, except for the last dense layer that uses the Softmax activation function.

### 3.4. Optimization Algorithms

Gradient descent is a first-order optimization process that iteratively adjusts a neural network's learnable parameters to minimize the loss. Generally, the gradient indicates the direction in which the loss function's change rate is the steepest.

The learning rate is the rate at which each learnable parameter is modified in the positive direction of displacement, with a step size in the negative direction that is appropriate. The equation below represents the update equation mathematically:

(1) $$\begin{array}{c} W=W-\eta *\frac{\partial L}{\partial W}, \end{array}$$

where *W* is the learnable parameter vector, *η* denotes the step size, and *L* denotes the loss function. The gradient descent algorithm contains three major variations, depending on how many data samples used for gradient computation: minibatch gradient descent (MBGD), stochastic gradient descent (SGD), and batch gradient descent (BGD). In the BGD technique, the loss function gradient is calculated for the whole training dataset, whereas in the SGD approach, a parameter update is performed for each training sample. In the MBGD algorithm, the entire dataset for training is partitioned into minibatches, and the parameters are changed for each minibatch. On the one hand, BGD causes sluggish training and superfluous calculations. On the other hand, SGD is faster, and although there are swings owing to frequent updates with large volatility, it is generally stable. Minibatch gradient descent has a lower variance of parameter updates than the other two methods, which might lead to more steady convergence.

We used stochastic gradient descent with momentum (SGDM). The SGDM technique has been extended to include stochastic gradient descent with momentum [37]. It takes past gradients into consideration in each dimension. The term momentum prevents undesirable oscillations and speeds up the algorithm's convergence.

## 4. Experimental Results and Discussion

### 4.1. Dataset Description

In our experiments, we used a sample of images taken from the benchmark databases. On the citrus disease image gallery dataset, the combined dataset (citrus images database of infested scale and plant village), and our own gathered image database, the suggested approach is evaluated. Citrus diseases including anthracnose, black spot, canker, scab, greening, and melanose were detected and classified using these datasets. The suggested method outperforms current techniques. Our databases are divided into two databases; first one for fruit disease image (FDI), and the second one for leaf disease image (LDI). The description of our two databases is shown in Table 1.

**Figure 3:**
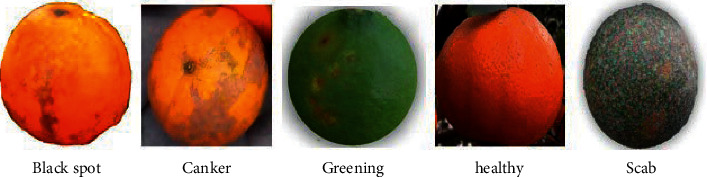
Sample of fruit disease image (FDI).

**Figure 4:**
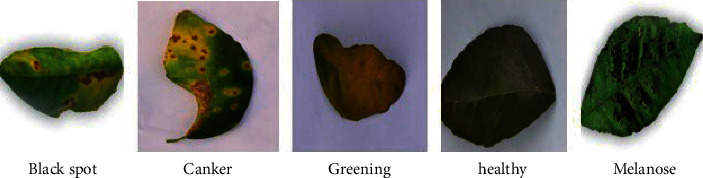
Sample of leaf disease image (LDI).

The samples of first database FDI and the samples of second database LDI are presented in Figure 3 and Figure 4.

### 4.2. Data Augmentation

To be successfully trained, deep learning classification networks require a large amount of training data. Unfortunately, the number and scarcity of current citrus disease image collections, as well as the lack of genuine annotated ground truths, continue to obstruct the automatic diagnosis of citrus diseases. To solve this problem, augmentation operations on the training set were performed to increase the number of training images and avoid the overfitting problem that can occur in the case of using a small amount of training data during the training process. Several augmentation parameters, such as random cropping, rotation, mirroring, and color shifting, were applied to the data using principal component analysis. After augmentation, we get 12,211 images, as illustrated in Table 2. The input images are transformed to a standard size of 256 ^∗^ 256 dimensions in the proposed work to make implementation easier and save processing time.

We employed different proportions of training and testing samples to evaluate the efficiency of the proportion of training and testing samples' number on classification. We used 80–20 and 60–40 training and testing samples, respectively. As the number of training samples grows, it is projected that the technique's accuracy would improve.

For the classification of citrus disease images, AlexNet and VGG19 networks are trained and evaluated using the two datasets. The pretrained model is divided into two phases; in the first phase, the dataset is split into 80% of the images for training and 20% for testing, and in the second phase, the dataset is split into 60% of the images for training and 40% for testing of the images for each database. SGDM optimizers are used for training. The following equations define the performance metrics utilized in this study.

The sensitivity, otherwise called recall, indicates the precision of positive instance, and it refers to how many examples of positive sets were correctly labelled; it can be measured using equation (2), where TP represents true positive or the numeral of positive cases that are precisely classified, and FN represents false negatives or the quantity of positive cases that are inaccurately named as negatives.

(2) $$\begin{array}{c} \mathrm{sensitivity}\left(\mathrm{recall}\right)=\frac{\mathrm{TP}}{\mathrm{TP+FP}}. \end{array}$$

Specificity is explained as the restrictive probability of actual negative token of an optional class, which generally corresponds to the likelihood of the negative marking which is true; it is expressed by equation (3), where TN signifies the quantities of cases or real negatives that are negative and named such true, and FP indicates the quantities of false upsides or cases that are erroneously delegated positive.

(3) $$\begin{array}{c} \mathrm{specificity}=\frac{\mathrm{TN}}{\mathrm{TN+FP}}. \end{array}$$

In general, sensitivity and accuracy measure the algorithm's effectiveness on a certain class and are either negative or positive, respectively.

Precision is the most frequent criterion for assessing categorization efficiency. During the assessment period, the accuracy is evaluated every 20 iterations. This metric, which counts the proportion of samples that are properly categorized, is represented by the following equation:

(4) $$\begin{array}{c} \mathrm{accuracy}=\frac{\mathrm{TP+TN}}{\mathrm{TP+TN+FP+FN}}. \end{array}$$

Equation (5) gives precision when we divide the number of true positives by the same number plus the number of false positives. This statistic assesses the algorithm's accuracy or its ability to anticipate results. The model's precision relates to how “exact” it is in terms of how many of the anticipated positives are actually positive.

(5) $$\begin{array}{c} \text{precision}=\frac{\text{TP}}{\text{TP}+\text{FP}}. \end{array}$$

The performance of a deep neural network may be improved by properly selecting hyperparameters such as batch size, maximum epochs, and step size. For training the pretrained models, a batch size of 32 is chosen. A low step size of 0.00001 and a number of epochs of 20 may lead to better network performance when transferring pretrained network weights. The SGDM optimizer is used to change network settings for each database, and performance measurements are recorded. The simulation findings show that when a pretrained model is trained with 80% of the images and tested with 20% of the images, the SGDM optimizer produces superior results.

Two different phases, 60 : 40 and 80 : 20, are used to calculate results. In the first stage, 60 : 40 approach is used to calculate classification performance for two datasets, and results are shown in Tables 3 and 4. They explain the findings achieved using the AlexNet and VGG19 models for various datasets. AlexNet classifier gives better classification accuracy as compared with VGG19 classifiers. On AlexNet, the achieved accuracy is 91.4%, while precision, sensitivity, specificity, and F-score are 90.7%, 90.6%, 90.4%, and 90.9%, respectively, but on VGG19, the achieved accuracy is 91.1%, while precision, sensitivity, specificity, and F-score are 90.1%, 90.3%, 90.2%, and 90.7%, respectively.

In the second stage, evaluation is performed by using a ratio of 80 : 20. Tables 5 and 6 illustrate the results of this phase for two datasets. They explain the findings achieved using the AlexNet and VGG19 models for various datasets. It can be seen that the AlexNet classifier gives better classification accuracy as compared with the VGG19 classifier. On AlexNet, the achieved accuracy is 93.5%, while precision, sensitivity, specificity, and F-score are 93.2%, 93.1%, 92.9%, and 93.4%, respectively, but on VGG19 the achieved accuracy is 92.6%, while precision, sensitivity, specificity, and F-score are 92.4%, 92.2%, 92%, and 92.5%, respectively.

**Figure 5:**
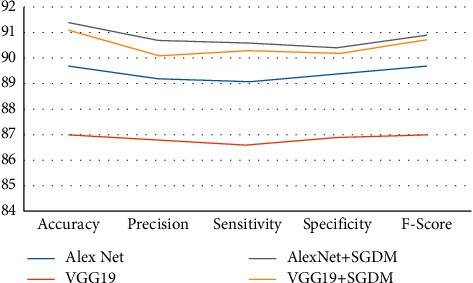
Proposed method classification performance for strategy 60 : 40 on LDI dataset.

**Figure 6:**
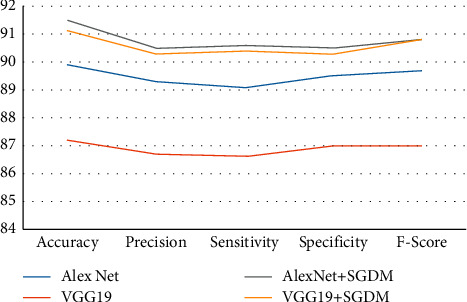
Proposed method classification performance for strategy 80 : 20 on LDI dataset.

**Figure 7:**
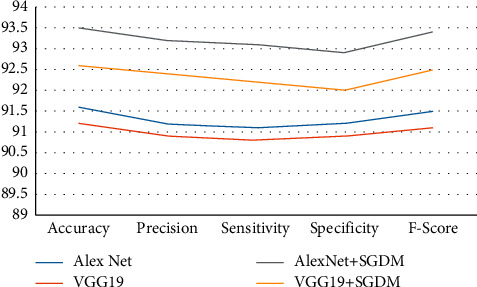
Proposed method classification performance for strategy 60 : 40 on FDI dataset.

**Figure 8:**
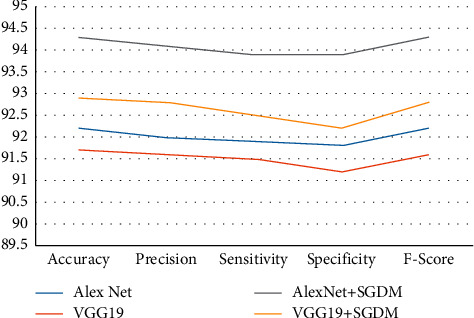
Proposed method classification performance for strategy 80 : 20 on FDI dataset.

The classification rate, accuracy, precision, sensitivity, specificity, and F-score of the proposed and current techniques are shown in Figures 5–8. In all experiments, the suggested technique outperforms the existing methods in terms of classification rate, accuracy, precision, sensitivity, specificity, and F-score.

The study results of Sharif et al. [38], dealing with of citrus diseases, were compared with the results of our study and are shown in Table 7. As seen in this table, the AlexNet with the SGDM model achieved high accuracy than studies of Sharif et al. with the original dataset.

## 5. Conclusion

The performance of SGDM optimizers for the automated identification of citrus disease images using the transfer learning approach is evaluated and compared in this paper. For extracting discriminative features from the source images, two standard models, AlexNet and VGG19, are examined. To assess network performance, the FDI and LDI citrus disease datasets are used to reach the greatest classification accuracy of 94.3%. Based on the findings, we determined that the deep learning methodology is a mature approach when compared to other methods. When we have a big amount of training data, we may also use an 80 : 20 strategy depending on the results. Data availability is considered to be the main hindrance of this work, which is reduced partly due to the incorporation of the data augmentation stage. In future research, we will concentrate on these flaws and work to enhance accuracy and classification algorithms.

## Figures and Tables

**Algorithm 1:**
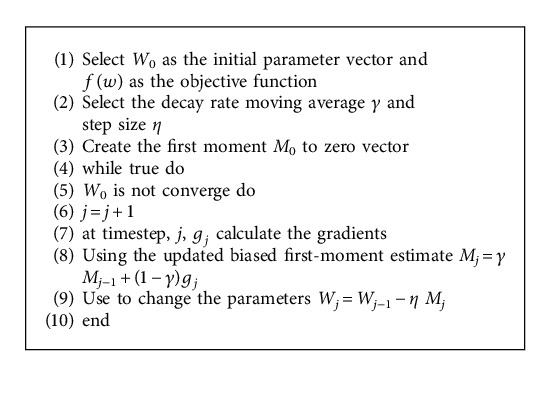
SGDM optimizer.

**Table 1:** Number of our two dataset samples.

Database name	Disease classes	Number of images
Fruit disease image (FDI)	Black spot	19
	Canker	78
	Greening	16
	Healthy	22
	Scab	15
Leaf disease image (LDI)	Black spot	171
	Canker	163
	Greening	204
	Healthy	58
	Melanose	13
Total images		759

**Table 2:** Number of our two dataset samples after augmentation.

Database name	Disease classes	Number of images	Number of images after augmentation
Fruit disease image (FDI)	Black spot	19	1,209
	Canker	78	1,678
	Greening	16	1,589
	Healthy	22	1,400
	Scab	15	1,590
Leaf disease image (LDI)	Black spot	171	1,031
	Canker	163	927
	Greening	204	1,007
	Healthy	58	800
	Melanose	13	980
Total images		759	12,211

**Table 3:** Proposed method classification performance for strategy 60 : 40 on FDI dataset.

Performance measures	Without SGDM		With SGDM	
	AlexNet (%)	VGG19 (%)	AlexNet (%)	VGG19 (%)
Accuracy	89.7	87	91.4	91.1
Precision	89.2	86.8	90.7	90.1
Sensitivity	89.1	86.6	90.6	90.3
Specificity	89.4	86.9	90.4	90.2
F-score	89.7	87	90.9	90.7

**Table 4:** Proposed method classification performance for strategy 60 : 40 on LDI dataset.

Performance measures	Without SGDM		With SGDM	
	AlexNet (%)	VGG19 (%)	AlexNet (%)	VGG19 (%)
Accuracy	89.9	87.2	91.5	91.1
Precision	89.3	86.7	90.5	90.3
Sensitivity	89.1	86.6	90.6	90.4
Specificity	89.5	87	90.5	90.3
F-score	89.7	87	90.8	90.8

**Table 5:** Proposed method classification performance for strategy 80 : 20 on FDI dataset.

Performance measures	Without SGDM		With SGDM	
	AlexNet (%)	VGG19 (%)	AlexNet (%)	VGG19 (%)
Accuracy	91.6	91.2	93.5	92.6
Precision	91.2	90.9	93.2	92.4
Sensitivity	91.1	90.8	93.1	92.2
Specificity	91.2	90.9	92.9	92
F-score	91.5	91.1	93.4	92.5

**Table 6:** Proposed method classification performance for strategy 80 : 20 on LDI dataset.

Performance measures	Without SGDM		With SGDM	
	AlexNet (%)	VGG19 (%)	AlexNet (%)	VGG19 (%)
Accuracy	92.2	91.7	94.3	92.9
Precision	92	91.6	94.1	92.8
Sensitivity	91.9	91.5	93.9	92.5
Specificity	91.8	91.2	93.9	92.2
F-score	92.2	91.6	94.3	92.8

**Table 7:** Comparison of deep learning methods for citrus disease classification.

Study	Accuracy (%)	Precision	Sensitivity	Specificity	F-score
Sharif et al.	89.0	—	—	—	—
Our model	94.3	94.1	93.9	93.9	94.3

## Data Availability

The data used to support the findings of this study are available from the corresponding author upon request.
